# Behaviour of Non-Donor Specific Antibodies during Rapid Re-Synthesis of Donor Specific HLA Antibodies after Antibody Incompatible Renal Transplantation

**DOI:** 10.1371/journal.pone.0068663

**Published:** 2013-07-26

**Authors:** Nithya S. Krishnan, Daniel Zehnder, Sunil Daga, Dave Lowe, F. T. Lam, Habib Kashi, Lam Chin Tan, Christopher Imray, Rizwan Hamer, David Briggs, Neil Raymond, Robert M. Higgins

**Affiliations:** 1 Department of Nephrology, University Hospitals Coventry and Warwickshire National Health Service Trust, Coventry, United Kingdom; 2 Department of Histocompatibility and Immunogenetics, National Health Service Blood and Transplant, Birmingham, United Kingdom; 3 Clinical Sciences Research Laboratory, Warwick Medical School, University of Warwick, Coventry, United Kingdom; 4 Division of Health Sciences, Warwick Medical School, University of Warwick, Coventry, United Kingdom; University of California Los Angeles, United States of America

## Abstract

**Background:**

HLA directed antibodies play an important role in acute and chronic allograft rejection. During viral infection of a patient with HLA antibodies, the HLA antibody levels may rise even though there is no new immunization with antigen. However it is not known whether the converse occurs, and whether changes on non-donor specific antibodies are associated with any outcomes following HLA antibody incompatible renal transplantation.

**Methods:**

55 patients, 31 women and 24 men, who underwent HLAi renal transplant in our center from September 2005 to September 2010 were included in the studies. We analysed the data using two different approaches, based on; i) DSA levels and ii) rejection episode post transplant. HLA antibody levels were measured during the early post transplant period and corresponding CMV, VZV and Anti-HBs IgG antibody levels and blood group IgG, IgM and IgA antibodies were quantified.

**Results:**

Despite a significant DSA antibody rise no significant non-donor specific HLA antibody, viral or blood group antibody rise was found. In rejection episode analyses, multiple logistic regression modelling showed that change in the DSA was significantly associated with rejection (p = 0.002), even when adjusted for other antibody levels. No other antibody levels were predictive of rejection. Increase in DSA from pre treatment to a post transplant peak of 1000 was equivalent to an increased chance of rejection with an odds ratio of 1.47 (1.08, 2.00).

**Conclusion:**

In spite of increases or decreases in the DSA levels, there were no changes in the viral or the blood group antibodies in these patients. Thus the DSA rise is specific in contrast to the viral, blood group or third party antibodies post transplantation. Increases in the DSA post transplant in comparison to pre-treatment are strongly associated with occurrence of rejection.

## Introduction

Antibodies are major factors not only in the human immune response against bacteria and viruses but also for allograft rejection and transplant kidney survival. The determinants of the levels of antibodies are not fully understood. Until recently it has been difficult to study the characteristics of human leukocyte antigen (HLA) antibodies after transplantation in the face of preformed HLA antibodies, first because the methods used to measure antibody levels were neither sensitive nor specific, and secondly because the results of such transplants were poor. It is now possible to follow the levels of HLA antibodies closely after renal transplantation.

There has always been a theoretical concern that infections can trigger rejection episodes and increase HLA antibodies. A recent study has shown that there is a strong association between the development of infection and increases in both breadth and strength of HLA antibodies [1]. The increase in the breadth of HLA antibodies was mainly due to expansion of reactivity among other antigens of a cross-reactive group (CREGs). Other studies have shown that in transplant kidney biopsies of acutely rejecting patients with viral infections the presence of plasma cell infiltrates and C4d deposition [2], [3]. The relationship between infection and rise in HLA antibodies is thought to be secondary to the presence of a robust memory B-cell response to the release of pro-inflammatory cytokines.

It is of interest to find out if rises in DSA levels with or without rejection is associated with rise in viral and blood group antibodies. The response of blood group and viral antibodies in pre-sensitized patients to a renal allograft is not fully understood. Changes in the levels of these antibodies soon after transplantation might illuminate the relationship between DSA and these antibodies and there may be insights into the processes determining the production and elimination of HLA antibodies [4].

With regards to blood group antibody levels after blood group incompatible transplantation, studies have shown a reduction in the levels of blood group antibodies in the long term, to undetectable levels in many patients. Higher levels of antibodies were associated with short-and long term dysfunction in some patients [5]. The rapid disappearance of blood group antibodies soon after transplantation in many patients with good functioning grafts contrasts with the reports in HLA antibody incompatible transplantation [6], [7], [8]. Looking at blood group antibody levels after HLA antibody incompatible transplantation would confirm whether the changes observed after blood group incompatible transplantation are specific to that setting, or also occur when there is a marked humoral response to HLA after transplantation.

The aims of this study were to examine in detail the wider humoral response during a period of intense re-synthesis of HLA antibodies after renal transplantation. The choice of antibodies studied meant we studied antibodies that had been stimulated by infection, immunization, and ‘natural’ antibodies.

## Methods

The study was approved by the West Midlands Research Ethics committee, U.K. Patients sensitized to HLA antigens were selected after obtaining consent for our program of antibody incompatible transplantation if they had current reactivity with donor specific HLA antigens measured by cytotoxic crossmatch (CDC), flow crossmatch (FC), or by microbead assay. We analysed 55 such patients, 31 women and 24 men, who underwent HLAi renal transplant at our center from September 2005 to September 2010. Pre-transplant, patients were treated with five alternate day sessions of double filtration plasmapheresis, the aim being to achieve a negative flow crossmatch at the time of surgery. In some cases with low starting levels of DSA, fewer sessions of plasmapheresis were administered. In some cases with high starting levels of DSA, more sessions of plasmapheresis were administered, and/or the transplant was performed in the presence of positive crossmatch. The number of plasmapheresis sessions administered varied between two to seven, with the majority getting five sessions. Patients who had blood group antibody incompatibility or who died in the early post-transplant period were excluded from our study.

Serum samples for antibody analysis were done at four time points, namely pre-treatment, at peak DSA post transplant, at rejection and late sample which was around six weeks to three months post transplant. Peak DSA was defined as the highest level of DSA within the first six weeks post transplant. As samples were collected from patients on a daily basis, the peak time point was chosen on retrospective analysis. Third party antibodies (TPA) were defined as HLA antibodies in the recipient, which were not specific for epitopes expressed on the donor antigens. Though there were many potential TPA’s, the one which was predominant in that individual patient, was studied.

### Immunosuppression

Imunosuppression consisted of mycophenolate mofetil 1000 mg bd started five days before transplant, with dose reduced if white cell count fell below 4.0×10^9^/l. Tacrolimus was started three days before transplant at a dose of 0.15 mg/kg/day in divided doses, with a target trough level of 10–15 µg/l in the first month. Prednisolone 20 mg od was started at the time of surgery, and methylprednisolone 500 mg was given as a single intravenous dose during the transplant operation. Two doses of basiliximab 20 mg were given, at days zero and four. The protocol was the same as stated in our previous publications [9], [10].

### Rejection

Rejection was diagnosed by renal biopsy if the renal function deteriorated, or clinically if there was rapid onset oliguria with a rise in both creatinine and in DSA levels. Biopsies were when clinically indicated and these were independently analysed by pathologists. Diagnosis of antibody mediated rejection, cellular rejection or mixed were made according to Banff classification of transplant biopsies [11], [12]. Rejection was treated with three days high dose methylprednisolone and OKT3 early on in the series or ATG later on. Rituximab was not given to any of our patients; two patients received IVIG one month post transplant and four patients received post-transplant plasmapheresis.

### Microbead Assays

The main DSA, cumulative DSA and the third party HLA Abs both HLA Class I and Class II specific antibodies were analysed using microbead assay manufactured by One Lambda Inc (Canoga Park, CA, USA), analysed on the Luminex platform (XMap 200, Austin, TX, USA) as used in similar studies previously [13]. Raw mean fluorescence intensity (MFI) values were used to follow antibody levels. All assays were performed using serum/bead volume ratios and one thousand MFI was used as the cut off for positive and negative beads according to the manufacturer’s instructions.

#### Flowcytometric estimation of blood group antibodies

Plasma samples were analysed using flowcytometry for estimating IgG, IgM and IgA blood group antibodies against reagent cells. We have used the method previously published by us [14].

#### Quantification of viral antibodies using LIAISON® analyzer

Using the LIAISON ® analyzer (DiaSorin S.p.A, Saluggia, Italy), Cytomegalovirus (CMV), Varicella Zoster (VZV) and Anti Hepatitis B Surface antigen (Anti-HBsAg) IgG antibodies were quantified from the corresponding serum samples according to manufactures instructions. The LIAISON® viral antibody test is a fully automated two-step direct sandwich immunoassay for in *vitro* quantitative determination of antibodies to the specific viral antigen, based on chemiluminescent technology, to be run on the LIAISON. The method for quantitative determination of specific IgG to viral antigen is an indirect chemiluminesence immunoassay (CLIA) [15].

#### CMV screening and prophylaxis

Routine CMV screening is done one, three and six months post transplant in all patients using quantitative PCR (Argene PCR, Biomerieux, CMV R- gene quantification assay) with the cut-off for positivity being 400 CMV copies/ml of plasma. As per protocol all CMV IgG negative (R-) recipients who receive CMV IgG positive (D+) kidneys and any recipient receiving ATG or OKT3 will get prophylaxis with Valganciclovir for three months. Primary CMV infection is defined as CMV viremia post transplant in a sero-negative recipient. Secondary infection is CMV viremia in a sero positive patient post transplant.

### Statistical Analysis

For comparison of baseline characteristics between groups, including rejection status defined, the chi-squared test was used for categorical variables. Means and their differences were compared using the t-test and Wilcoxon non-parametric test as appropriate and the level of significance was set at P<0.05.

To investigate the influence of antibody levels and changes in levels from pre-transplant to post transplant peak on the risk of rejection, allowing for the effects of other antibodies and potentially confounding variables, multiple logistic regression modelling was used. In all logistic models, age, sex, DR mismatch and number of previous transplants were retained as potentially confounding. The combined DSA (classes I & II) antibodies levels, pre-transplant, post transplant peak and change (peak – pre) were investigated as the main explanatory factor for rejection. Pre-transplant, peak and changes in other antibodies were included in models, to examine their influence on the DSA antibody effects.

IBM SPSS software version –19 was used to compare the antibody levels between the groups. The overall trend over time was analysed using Kendall test.

## Results

We analysed the data using two different approaches; i) based on DSA levels and ii) based on rejection episode post transplant. Since the primary study aim was to see if non-HLA antibody levels increased along with the HLA antibodies, the patients were divided into 2 groups; 1) Group 1 - Patients who had higher post transplant peak DSA than pre-treatment levels and 2) Group 2 - those that had lower post transplant peak DSA than pre-treatment levels. Patient characteristics are summarized in Table 1. There were 34 patients in Group 1 and 21 in Group 2. The mean age was similar in both the groups (38.9 and 40.9). The mean and the range (given in parentheses) pre-treatment class I & II DSA were 3875 (220, 9181) and 5317 (331, 11055) in Group 1 and 7847 (799, 60605) and 7281 (315, 19652) in Group 2 respectively. And the mean peak class I & II DSA post transplant were 7024 (193, 12383) and 7195 (514, 12811) in Group 1 and 4449 (120, 15914) and 5757 (447, 27756) in Group 2 respectively. Two patients received IVIG one month post transplant and four patients received post-transplant plasmapheresis. Analysis was repeated excluding these patient samples and there was no difference in the results.

**Table 1:** Patient Characteristics.

		Groups N = 55			Rejection status N = 52		
		Peak DSA higher - Group 1 (n = 34)	Peak DSA lower - Group 2 (n = 21)	P value	Rejection(n = 26)	No Rejection(n = 26)	P value
**Male**		13 21	11 10	**0.3041**	10 16	12 14	**0.5745**
**Female**							
**Age (mean)**		44.8	43.8	**T = 0.31, p = 0.761**	45.5	42.0	**T = −1.07 p = 0.287**
**Number of previous transplants**	**0 1 2 3**	12 17 3 2	6 11 2 2		9 13 1 3	8 13 4 1	NS
**DR mismatch**	**0 1 2**	6 23 5	7 12 2		4 16 6	9 16 1	NS
**Change in DSA with reference to rejection Negative Positive**					6 20	17 9	**0.0021**
**Change in TPA with reference to rejection Negative Positive**					17 9	15 11	NS
**Rejection with reference to peak DSA Yes No**		23 9	3 17				**0.0001**

DSA- Donor specific antibody; TPA – Third party antibody.

Though some of the patients were negative for CMV IgG, anti- HBsAg or VZV IgG pre- transplant, they were included in the study. This is because we measured the actual values to obtain a continuous data to see if there was a rise in the level of these antibodies along with the DSA’s or not. If the patients were negative at pre-treatment and stayed negative throughout the four time points, they were excluded from the analysis, as it was not possible to differentiate between a lack of response and a lack of prior immunization. Thus in Group 1, 25 patient samples were analysed for CMV; 32 for VZV and 23 for anti- HBsAg. Similarly in Group 2, 13 patient samples were analysed for CMV, 20 for VZV and eight for anti-Hbs.

When measuring the viral antibodies, if the samples had reached the saturation of the assay, they were retested in 1 in 10 dilutions. There was no increase detected in the viral antibody level even on dilutions. Five patients had CMV viremia post transplant. Two of them had primary infection with concomitant change of CMV antibodies from negative pre-treatment to positive in late samples. Out of the three who had secondary infection one had no change in antibodies whereas the other two showed a slight increase in the late samples. Analysis was repeated excluding these patient samples and there was no difference in the results.

Thirty two patient samples from group 1 and 20 from group 2 were analysed for blood group antibodies and rejection outcome. There were three patients in total who were excluded for analysis of blood group antibodies from the two groups, as they had received both blood group incompatible and HLA incompatible kidney transplantation. Twenty three out of 32 patients had an episode of rejection in group 1 as opposed to three out of 20 in group 2, which was statistically significant (p = 0.0001). Fourteen patients had antibody mediated rejection, five had cellular rejection, two had mixed and five were treated as rejection clinically though the biopsies did not show evidence of rejection.

**Figure 1:**
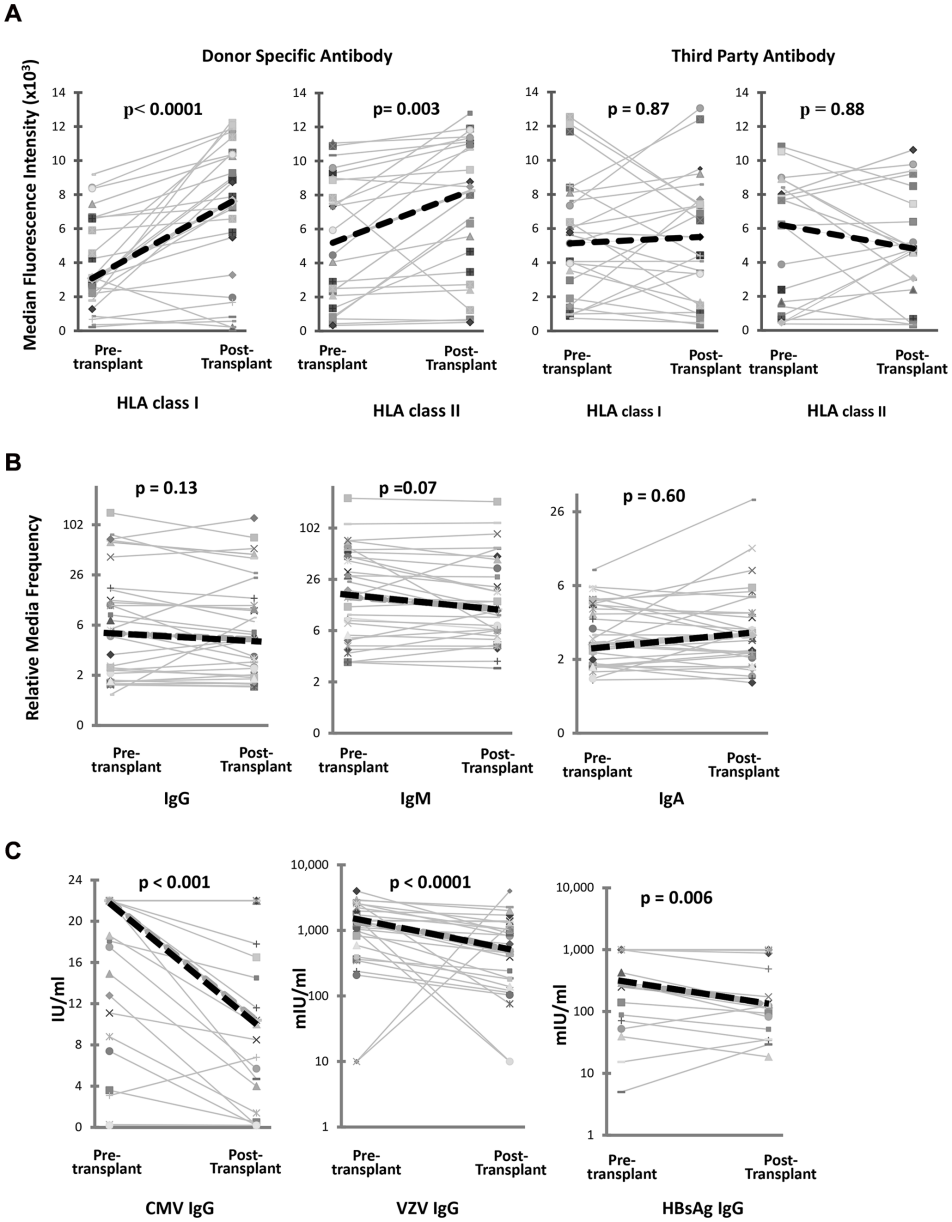
Antibody response in patients with an acute rise in donor specific HLA antibody after HLA antibody incompatible renal transplant. This shows the changes in **A)** the donor kidney specific antibody (DSA) for HLA class I & II and third party class I & II, **B)** IgG, IgM and IgA blood group antibodies and **C)** viral antibody levels in patients with significantly higher post-transplant peak DSA levels compared to pre-transplant levels. There was no rise in third party HLA antibodies or blood group antibodies. The viral antibodies showed a significant fall in serum antibody levels; cytomegalovirus (CMV) IgG (p<0.001), varicella zoster virus (VZV) IgG (p<0.0001) and Hepatitis B surface antigen (HBsAg) IgG antibody (p = 0.006). Only patients with measurable viral antibody levels pre-transplant were included. Graphs show individual patients (solid thin lines). Mean values are illustrated with the thick dashed line.

There was a statistically significant increase in the DSA levels between pre-treatment and peak level post transplant in class I and class II antibodies in group 1. This was associated with a simultaneous statistically significant decrease in the viral titres, but no change in blood group antibodies. With regards to the third party antibodies, though class I showed an increasing trend this was not statistically significant. Figure 1 shows the changes in the DSA class I and II, third party class I and II, viral and blood group antibody levels in group I patients, comparing pre-treatment levels to peak DSA levels.

**Figure 2:**
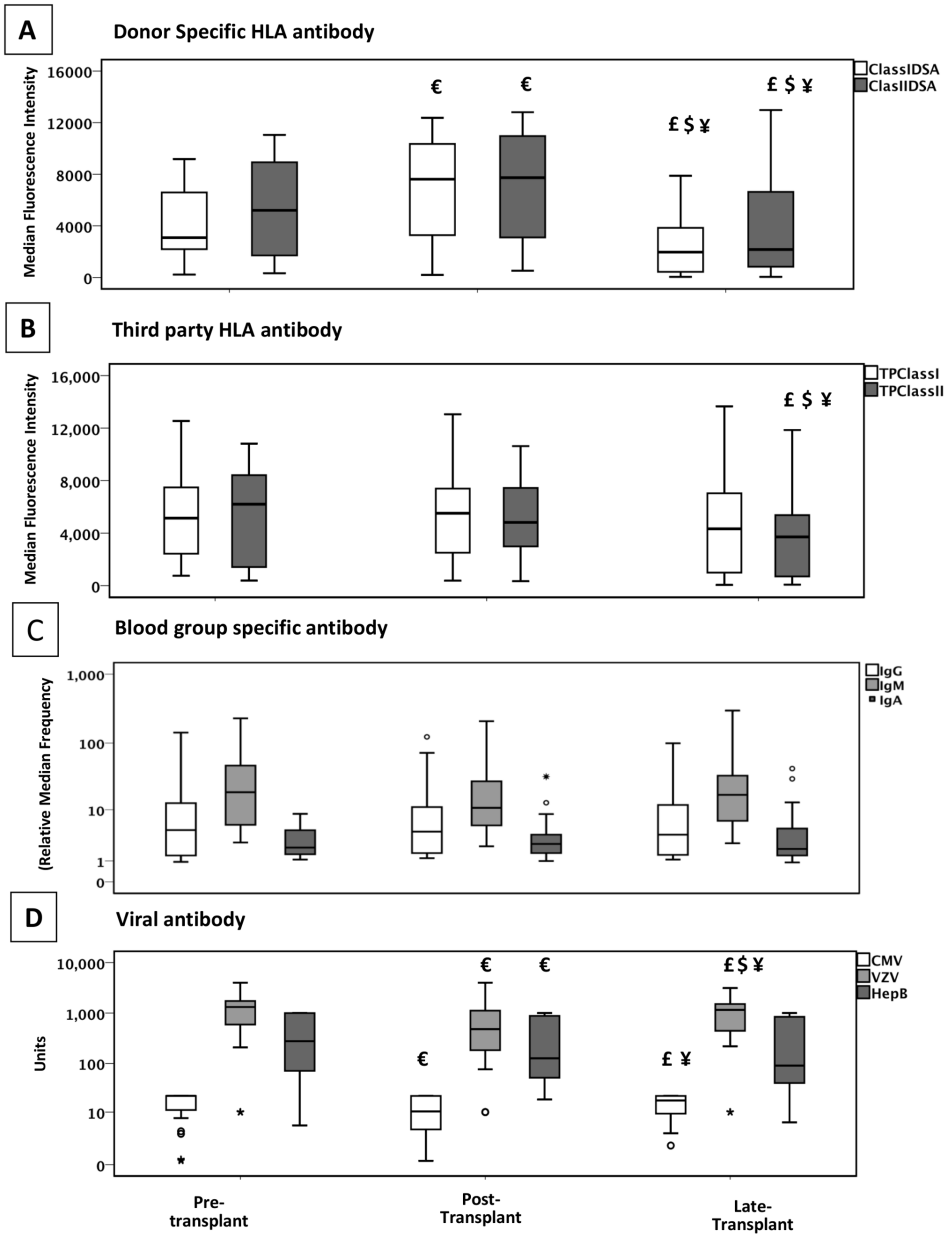
Antibody response during the first few weeks after HLA antibody incompatible renal transplant in patients with an acute rise in donor specific HLA antibody. This shows the changes in **A)** the donor kidney specific antibody (DSA) for HLA class I and II, **B)** third party class I and II, **C)** blood group antibodies and **D)** viral antibody levels in patients with significantly higher post-transplant peak DSA levels compared to pre-transplant levels over the first couple of months. Over a longer observation period there was no significant change in third party HLA antibody, IgG, IgM or IgA blood group antibody, cytomegalovirus (CMV) IgG, varicella zoster virus (VZV) IgG and Hepatitis B surface antigen (HBsAg) IgG antibody observed. Only patients with measurable viral antibody levels pre-transplant were included. Box plot shows the statistical significant changes in the groups €- p<0.05 pre-transplant vs. post-transplant; £ - p<0.05 post-transplant vs. late; $ - p<0.05 pre-transplant vs. late and ¥ - p<0.05 overall trend.

**Figure 3:**
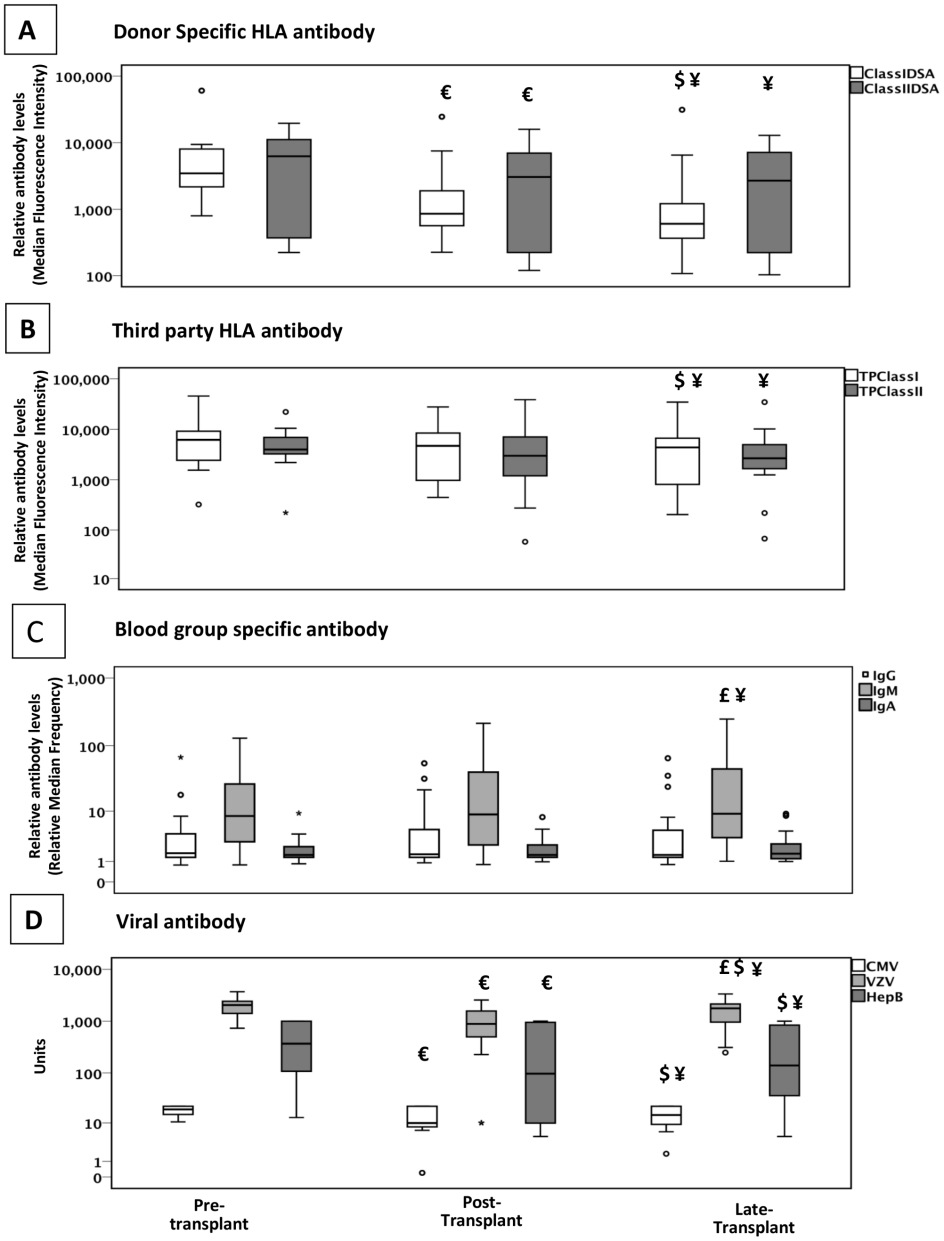
Antibody response during the first few weeks after HLA antibody incompatible renal transplant in patients with an acute fall in donor specific HLA antibody. This shows the changes in **A)** the donor kidney specific antibody (DSA) for HLA class I and II, **B)** third party class I and II, **C)** blood group and **D)** viral antibody levels in patients with significantly lower post-transplant peak DSA levels compared to pre-transplant levels over the first couple of months. Over a longer observation period there was no significant change in third party HLA antibody, IgG, IgM or IgA blood group antibody, cytomegalovirus (CMV) IgG, varicella zoster virus (VZV) IgG and Hepatitis B surface antigen (HBsAg) IgG antibody observed. Only patients with measurable viral antibody levels pre-transplant were included. Box plot shows the statistical significant changes in the groups €- p<0.05 pre-transplant vs. post-transplant; £ - p<0.05 post-transplant vs. late; $ - p<0.05 pre-transplant vs. late and ¥ - p<0.05 overall trend.

In group 2, there was a statistically significant fall in the DSA levels between pre-treatment and peak post transplant. The fall in the viral titres was also significant, but there was no change in the blood group antibody levels. Figures 2 and 3 show the trend of the different antibodies over time points of pre-treatment, peak post transplant and late samples in group 1 and group 2 respectively.

**Figure 4:**
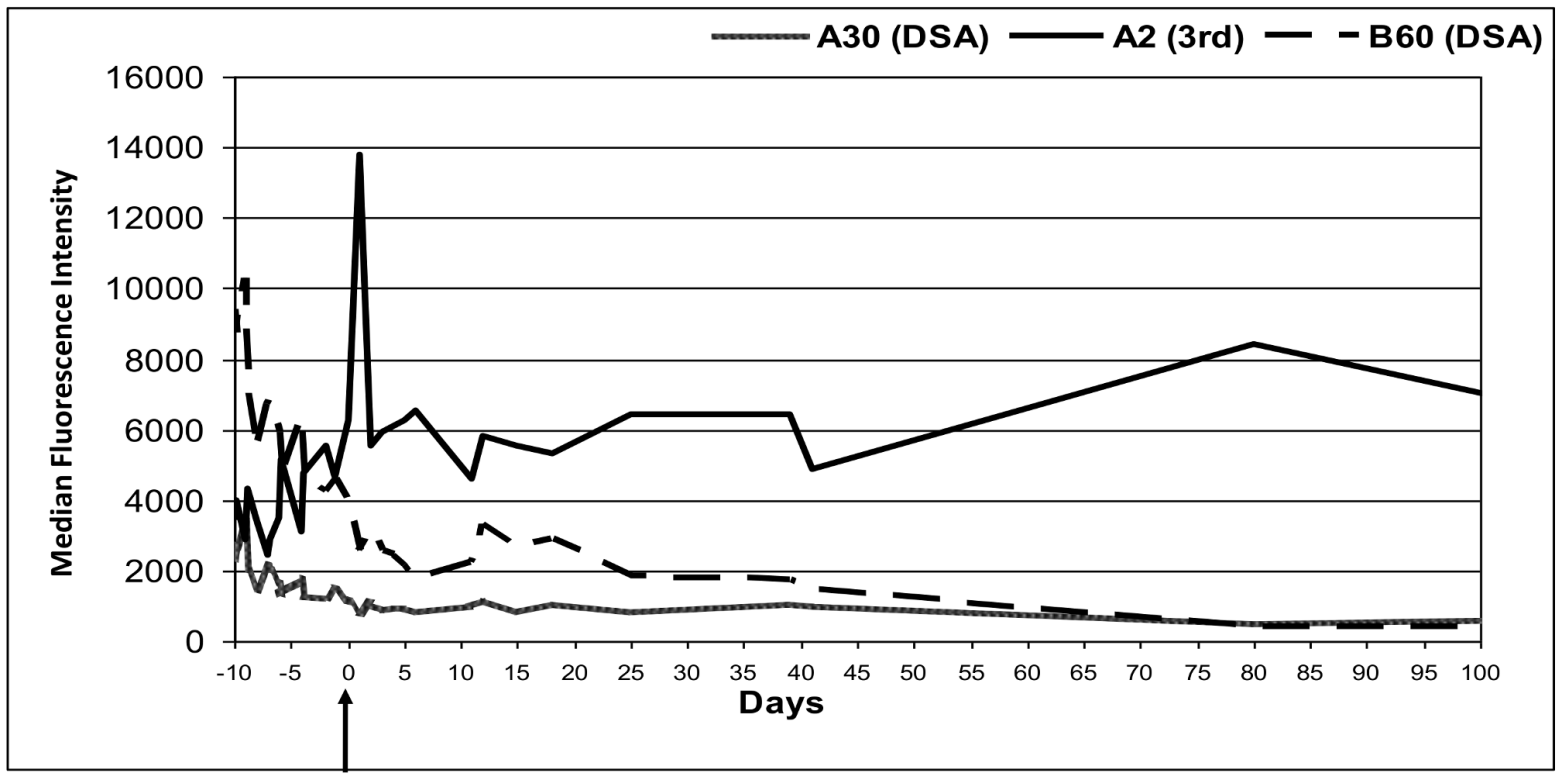
Patient with rise in third party HLA antibody after an HLA antibody incompatible renal transplant. Exceptionally a change in third party HLA antibodies was not noted which was not explained by the current understanding of epitope sharing of the third party HLA with donor specific HLA. In this example donor-specific antibodies to HLA A30 and B60 were going down post transplant, but the third party antibody HLA A2 was increasing. Though the HLA A2 is known to share epitopes with HLA A30, the behaviour of these two antibodies was very different to each other. Also, the patient did not receive any blood products after the transplant.

Though most of the rise or fall in class I third party antibodies could be attributed to shared epitopes with the DSA, some third party antibodies behaved completely different to the DSA. This cannot be explained by the current understanding of epitope sharing of the third party HLA with donor specific HLA. Figure 4 shows a patient wherein the DSAs HLA A30 and B60 were going down post transplant, but the third party antibody HLA A2 was rising and the patient did not receive any blood products after the transplant. In four other patients there was similar dissociation between donor specific and third party HLA antibody levels.

The second sets of analyses were done on 52 patients (excluding three as alluded to above) with reference to rejection as the outcome. Multiple logistic regression modelling was used to compare the occurrence of rejection with factors age, gender, DR mismatch, number of previous transplants, and DSA, TPA, viral and blood group antibodies (Table 2). Baseline levels of all variables including DSA were not predictive of rejection. This was true both in the crude model with no adjustments and in the ones following adjustment for age, sex previous transplant, DR mismatch and other baseline antibody levels. No other variables except change in the DSA were significantly associated with rejection (p = 0.01) consistently in all models with or without adjustment (all data not shown). An increase in DSA of 1000 units from pre treatment to a post transplant peak was equivalent to an increased odds of rejection of 30%, 1.30 (1.06, 1.58) if the adjustment was made for other antibodies at baseline. The odds increased to 50%, 1.47(1.08, 2.00) if it was adjusted for changes in the level of all other antibodies from pre to peak post transplant. Though VZV, anti Hbs and DR mismatch showed significance on isolated models, the effect was not constant.

**Table 2:** Results of multiple logistic regression modelling, comparing the occurrence of rejection in relation to DSA.

	Baseline pre-transplant DSA		Changes in DSA(Peak to Pre)		Changes in DSA(Peak to Pre)	
	Model 1		Model 2		Model 3	
	OR (95% CI)	P-value	OR (95% CI)	P-value	OR (95% CI)	P-value
**Any DSA pre-Tx (1000s)** #	1.08 (0.93, 1.25)	**0.2989**	1.30 (1.06, 1.58)	**0.0116**	1.47 (1.08, 2.00)	**0.015**
**Sex (F vs. M)**	1.32 (0.22, 7.95)	**0.7613**	0.58 (0.08, 4.54)	**0.6067**	0.45 (0.03, 6.56)	**0.5593**
**Age**	1.05 (0.98, 1.13)	**0.178**	1.07 (0.98, 1.18)	**0.141**	1.05 (0.96, 1.14)	**0.2964**
**Previous Tx (Yes vs. No)**	2.49 (0.36, 17.03)	**0.3532**	2.57 (0.27, 24.4)	**0.4111**	1.00(0.07, 15.08)	**0.997**
**DR mismatch (0 vs 1 or 2)**	3.78 (0.65, 21.93)	**0.138**	7.48 (0.85, 65.7)	**0.0696**	29.34 (1.96, 440.0)	**0.0145**
**Any TPA pre-Tx.**	1.00 (0.98, 1.02)	**0.8166**	1.00 (0.98, 1.02)	**0.9205**	1.00 (0.97, 1.03)	**0.7664**
**VZV pre-Tx.**	0.44 (0.20, 0.96)	**0.038**	0.37 (0.16, 0.84)	**0.0172**	2.32 (0.67, 8.05)	**0.1853**
**CMV pre-Tx.**	0.95 (0.87, 1.03)	**0.1869**	0.93 (0.85, 1.02)	**0.1031**	0.81 (0.66, 1.01)	**0.0623**
**antiHBsAg pre-Tx.**	1.00 (1.00, 1.00)	**0.3648**	1.00 (1.00, 1.00)	**0.2416**	1.02 (1.00, 1.03)	**0.0346**
**IgA pre-Tx.**	0.80 (0.51, 1.27)	**0.349**	0.69 (0.43, 1.11)	**0.1249**	0.92 (0.62, 1.37)	**0.689**
**IgG pre-Tx.**	1.02 (0.98, 1.06)	**0.4639**	1.02 (0.98. 1.07)	**0.3514**	1.04 (0.97. 1.11)	**0.2683**
**IgM pre-Tx**.	1.02 (0.99, 1.05)	**0.2334**	1.02 (0.99, 1.06)	**0.1917**	0.90 (0.80, 1.01)	**0.0773**

Model 1 is for DSA baseline level adjusted for age, sex, DR mismatch (0 vs. 1 or 2), previous transplant (Tx) (Yes vs. No), and baseline levels of TPA, CMV, VZV, IgA, IgG and IgM antibodies.

Model 2 is for change in DSA level (peak – pre-transplant), adjusted for age, sex, DR mismatch (0 vs. 1 or 2), previous Tx (Yes vs. No) and baseline levels of TPA, CMV, VZV, IgA, IgG and IgM antibodies.

Model 3 is for change in DSA level (peak – pre-transplant), adjusted for age, sex, DR mismatch (0 vs. 1 or 2), previous Tx (Yes vs. No) and changes in all antibody levels (peak – pre-transplant levels of TPA, CMV, VZV, IgA, IgG and IgM antibodies).

# In all models, DSA pre-transplant and change levels have been expressed in 1000s, so the OR presented are for an increase of 1000 DSA units.

## Discussion

The particular stimulus to this study was the observation that HLA antibodies may rise after an infection or blood transfusion, even though the HLA antigen is not being directly represented to the subject [1]. This raised important questions about the specificity of the humoral immune response. Hypotheses to explain the increase in HLA antibody levels during a viral infection include cross reactivity between epitopes on HLA molecules and viruses, immune up regulation secondary to a polyclonal antibody response to a single antigen, or an increase in non-self HLA expression on any non-self cells in the host expressing HLA [16]. One way interrogate these hypotheses was to look at the reverse situation in clinical practice, ie, during a response when the subject is synthesising HLA antibodies rapidly, do other antibody levels change?

This study has examined in detail, for the first time, the levels of HLA and several non-HLA antibodies after HLA antibody incompatible transplantation. The study has shown several novel findings. First, although HLA antibodies may rise during a viral infection, viral antibodies did not rise during an intense HLA antibody response. Second, there were some unexplained antibody responses to non-donor HLA antibodies, not explained by the current understanding in epitope sharing. On regrouping the patients with reference to rejection as the outcome and studying the relationship of all the variables, it was evident that the increase in the DSA peak post transplant in comparison to the pre-treatment level was the only factor significantly associated with occurrence of rejection.

Viral and blood group antibodies did not rise during intense re-synthesis of HLA antibodies, indicating specificity in the immune response and the lack of a bystander effect between plasma cell clones. Studies have shown that vaccination expands both specific and bystander memory T cells but antibody production remained vaccine specific [17]. Some other studies have shown that influenza vaccine in stable kidney transplant patients is not associated with the risk of acute rejection or increase in DSA levels [18], [19]. In contrast, other studies have shown vaccination to potentiate allograft rejection [20]. This is thought to occur due to non-specific immune activation and induction of cross-reactive immunity, resulting in enhanced humoral or cellular responses against the donor antigens [21], [22], [23]. A recent study from Switzerland showed that multiple doses of influenza vaccine may lead to the production of anti-HLA antibodies in a significant proportion of kidney transplant recipients [24].

We compared quantitative antibody analysis of latent viral antigens at different time points, to see if there was a rise in these antibodies due to non-antigen specific stimulation i.e. bystander activation of memory cells due to antigenically unrelated activation. This study was not designed to look at the dominant type of immune response (humoral and cellular) generated for controlling specific viral infections. A recent study looked at the longitudinal quantitative analysis of antibody titres specific for various viral antigens including varicella-zoster virus for a period of up to 26 years [25]. They showed that in spite of vaccinations, viral infections, and reactivation the antibody changes were very specific, thus ruling out bystander activation as a cause of antibody production.

Zachary and associates have previously shown that in HLA incompatible renal transplantation, the viral antibodies detected by ELISA did not change in relation to plasmapheresis [26]. It could be that the immunosuppressive medications could have profoundly decreased the humoral immune responsiveness [27]. However, in these patients the concomitant administration of cytomegalovirus immune globulin (CMVIg) with high levels of CMV antibodies meant that the samples used for testing were some weeks after the transplant. The timing of testing was based on calculated time for clearance of the CMVIg originated antibodies. This is the first study that has been able to examine the memory immune response early after transplantation, particularly during an intense donor-specific humoral response.

In our study patients received pre transplant plasmapheresis to remove DSA prior to transplantation. Also some patients received OKT3 or ATG for treatment of humoral and steroid resistant rejection post transplant. This could have modified all antibody levels post transplant. However, more than half of the study patients (29/52) had a significant rise in DSA levels post transplant, out of whom 18 patients received ATG/OKT3 for treatment of rejection. Thus it can be said that in spite of the overall immunosuppression there was an exquisite rise in the HLA antibodies and hence did not affect the analyses.

With regards to TPAs, many of these did follow the donor specific antibody levels, and this could be explained by epitope sharing. Because of the extent of possible epitope sharing it was not easy to exclude epitope sharing as the reason for change in non-donor specific HLA antibody levels in nearly all cases. However, in five cases there was evidence of a response in non-donor specific HLA antibody levels that was not explicable by any possible epitope sharing. It is possible that this observation is due to shared epitopes not previously described or another form of shared antigenicity such as denaturing of antigen on the microbeads. This requires further investigation with techniques such as absorption studies using donor specific HLA protein.

There are some shortcomings in this study. Samples were collected prospectively for studies on antibodies on a daily basis in the early post transplant period, so that changes in antibody levels were closely tracked. However, the inevitable heterogeneity in a clinical series means that patients did receive different treatments. Also, a more qualitative analysis like mapping of viral epitopes and the change over time, would aid in a better understanding of the infection-alloimmunity connection. Unfortunately this was outside the scope of this study. Further studies are needed in this area to increase our understanding about the immunological response.

## Conclusions

During a period of intense re-synthesis of donor specific HLA antibodies, it was possible to follow in detail the levels of non-donor specific HLA antibodies, and other antibodies previously stimulated by infection, immunization, or ‘natural’ blood group antibodies. Our results showed that the immune response was generally specific for donor HLA in contrast to the viral, blood group or third party antigens post transplantation and the increase in the DSA post transplant in comparison to pre-treatment is strongly associated with occurrence of rejection.
